# Health inequalities in Germany: do regional-level variables explain differentials in cardiovascular risk?

**DOI:** 10.1186/1471-2458-7-132

**Published:** 2007-07-01

**Authors:** Juergen Breckenkamp, Andreas Mielck, Oliver Razum

**Affiliations:** 1 Department of Epidemiology and International Public Health, School of Public Health, University of Bielefeld, POB 10 01 31, 33501 Bielefeld, Germany; 2 Institute of Health Economics and Health Care Management, GSF – National Research Center for Environment and Health, POB 11 29, 85758 Neuherberg, Germany

## Abstract

**Background:**

Socioeconomic status is a predictor not only of mortality, but also of cardiovascular risk and morbidity. An ongoing debate in the field of social inequalities and health focuses on two questions: 1) Is individual health status associated with individual income as well as with income inequality at the aggregate (e. g. regional) level? 2) If there is such an association, does it operate via a psychosocial pathway (e.g. stress) or via a "neo-materialistic" pathway (e.g. systematic under-investment in societal infrastructures)? For the first time in Germany, we here investigate the association between cardiovascular health status and income inequality at the area level, controlling for individual socio-economic status.

**Methods:**

Individual-level explanatory variables (age, socio-economic status) and outcome data (body mass index, blood pressure, cholesterol level) as well as the regional-level variable (proportion of relative poverty) were taken from the baseline survey of the German Cardiovascular Prevention Study, a cross-sectional, community-based, multi-center intervention study, comprising six socio-economically diverse intervention regions, each with about 1800 participants aged 25–69 years. Multilevel modeling was used to examine the effects of individual and regional level variables.

**Results:**

Regional effects are small compared to individual effects for all risk factors analyzed. Most of the total variance is explained at the individual level. Only for diastolic blood pressure in men and for cholesterol in both men and women is a statistically significant effect visible at the regional level.

**Conclusion:**

Our analysis does not support the assumption that in Germany cardiovascular risk factors were to a large extent associated with income inequality at regional level.

## Background

It is well established that employment grade, educational level, and household income are important predictors of mortality [1], cardiovascular risk factor levels and morbidity [2,3]. The international research supports an inverse association between socioeconomic status and cardiovascular disease [4-6]. More recently, the impact of socioeconomic factors throughout life course has been examined [7,8].An ongoing debate in the field of inequality and health focuses on two as yet unproven extensions of this association, which can be phrased as research questions:

1. Is individual health status associated with individual income and (particularly) with income inequality at aggregate (e. g. regional) level? [9]

2. If there is indeed an association between income inequality and health status, does it operate via a psychosocial pathway (stress due to perceptions of relative disadvantage and the psychological consequences of inequality) [9,10]; or via a „neo-materialistic" pathway (systematic under-investment across a wide range of societal infrastructures such as libraries, schools, hospitals)? [11]

Evidence for an association between income inequality and mortality at regional level has mostly come from the US [12,13]. No such association has been found in Canada and in Denmark [13,14], and Mackenbach (2002) considered the evidence to be "disappearing" and the US as "the exception" [15].

The debate is not over yet. The Danish study [14] was restricted to the capital, Copenhagen, a city that probably has better societal infrastructure than other parts of the country. Hence, the geographical unit used may have been too small [16] to conclude that there is no association between area income inequality and mortality in Denmark. Moreover, in the Scottish Heart Health Study, a significant variance in mean levels of cardiovascular risk factors persisted at the district level [17]; this again shows that factors related to area/place do influence health status.

A US study on cardiovascular disease risk factors found a contextual effect of income inequality for three of the four analyzed risk factors, notably among persons with low income [18]. Furthermore, in white Americans an inverse association of socioeconomic status with cardiovascular mortality was reported [19]. It has to be stated that, from a theoretical as well as from an empirical view, it is not clear which regional level is the most appropriate to analyze the question, but "... ignoring the role of group- or-macro-level variables may lead to an incomplete understanding of the determinants of disease in individuals as well as in populations" [20].

Ecologic and multilevel studies as well as comparisons of well defined areas have been conducted to investigate area effects. Multilevel models negotiate the restrictions of ecological studies (aggregate level). Area and individual level factors are analyzed simultaneously with the person as unit of analysis [21]. Most often, cross-sectional data are analyzed, providing a one-point measure of the association of interest. This does not take into account area effects in early life as origin of disease. For this purpose, a longitudinal study design (birth cohorts, record linkage) would be essential, focusing on the development of e.g. cardiovascular disease in later life.

The debate on income inequality and health should be pursued because of its obvious implications for public health policy. Findings could inform decisions as to the level at which interventions should be prioritized – at the individual level or at societal level. More studies are needed from outside the US.

Studies should be designed so they allow adjusting for income (or a proxy thereof, e. g. educational attainment) at individual level. Alternatively, a regional deprivation score, e.g. the Townsend Score, can be used as a measure [22].

Health status could be measured in terms of cardiovascular risk factor levels instead of mortality (requiring larger sample sizes/longer observation) or self-reported health (less reliable).

As the issue is to test hypotheses about different explanatory models, rather than to report on the present situation in a particular country, the data used for such analysis do not necessarily have to be recent. We here investigate the association of cardiovascular health status with income inequality at area levels controlling for individual socio-economic status for Germany. Admittedly, our data base does not allow for an investigation of the development of health inequalities during the past 25 years, such as has been influenced by reunification, migration of labor from east to west, (temporary) unemployment, flexible work arrangement, and fixed-term contracts, among others. However, this is not the primary objective of this study.

## Methods

**Figure 1:**
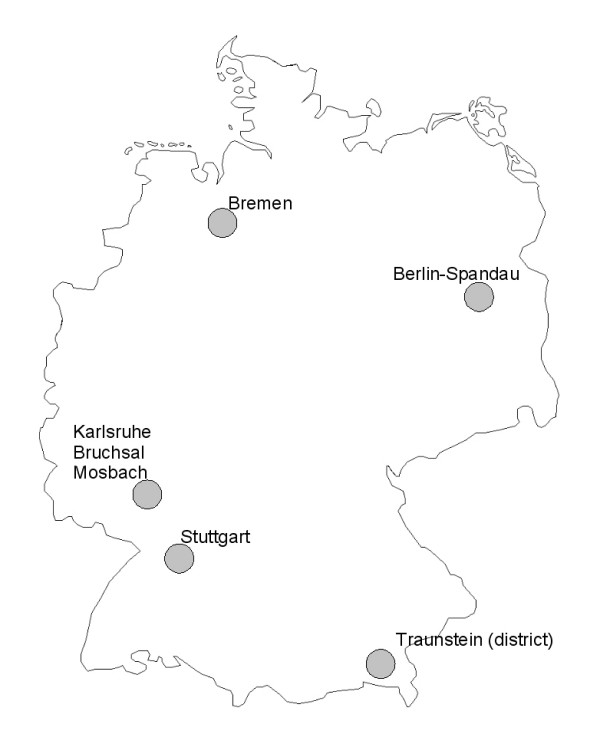
Intervention regions of the German Cardiovascular Prevention Study.

Appropriate individual-level exposure and outcome data are available from the baseline data set of the German Cardiovascular Prevention Study (DHP; 1984–1986), a cross-sectional, community-based, multi-center intervention study [23]. It comprises data from six geographically defined, socio-economically diverse intervention regions, covering a total population of about 356,000 persons (see figure 1). In each region, samples were drawn from registration offices, and about 1800 individuals aged 25 to 69 years possessing German citizenship were enrolled using a cluster sampling approach. Participation rates lay between 69.6 % and 82.5 %. For data analysis, one of the regions was split into two, because it comprises two separate cities, Bruchsal and Mosbach, with possibly different levels of inequality and risk factors. Therefore, the following regions were available for modeling analyses: Berlin-Spandau, Bremen North and West, Bruchsal, Karlsruhe, Mosbach, Stuttgart West and Vaihingen and the district of Traunstein. Regions differ considerably regarding gross value added per inhabitant, poverty rate and, to a lower degree, regarding unemployment (table 1).

**Table 1:** Cardiovascular risk factors and social status variables by region

			cardiovascular risk factors (means)								social status variables						
	N		BMI (kg/m^2^)		Diastolic blood pressure (mm/Hg)		Systolic blood pressure (mm/Hg)		Cholesterol (mg/dl)		Lower SES (in %)		Unemployment rate ^a^	Ginis ^b^	Gross-value added per inhabitant (DM), 1988	Poverty rate	
	men	women	men	women	men	women	men	women	men	women	men	women	(both)	(both)	(both)	men	women

Region 1	850	880	26.1	25.7	82.5	80.3	136.6	134.2	231.1	233.7	20.4	37.3	5.90	0.20658	42,800	12.4	16.1
Region 2	831	955	26.2	25.5	85.0	81.5	134.1	129.5	240.5	238.0	15.9	24.9	3.08	0.20773	34,900	4.2	6.8
Region 3	893	999	26.4	25.9	79.4	75.6	129.7	124.8	229.2	229.0	24.1	37.8	3.12	0.21976	28,100	20.4	21.6
Region 4	316	345	26.8	25.7	85.6	81.8	138.6	135.1	230.1	230.1	15.5	29.0	3.18	0.21634	23,900	10.4	14.2
Region 5	910	959	26.1	25.2	86.3	81.3	135.8	129.8	232.4	233.0	11.9	22.2	2.84	0.22173	26,500	7.1	10.1
Region 6	778	929	25.7	24.7	85.2	80.4	134.7	128.3	232.8	234.5	10.8	20.2	2.11	0.22323	66,000	7.3	7.5
Region 7	656	719	26.9	26.3	86.6	82.1	137.7	131.2	236.5	234.9	15.7	38.7	2.84	0.21292	23,900	10.1	16.1
Overall	5234	5786	26.3	25.5	84.1	80.2	134.9	129.8	233.4	233.5	16.5	29.8	3.31	0.21886	36,330	10.4	13.1

a) as reported by participants

b) a coefficient of 0.05 to 0.1 corresponds to a normal distribution of household income

All participants underwent medical examination. During examination, two blood pressure readings were taken. Results from the second reading and fifth phase (diastolic blood pressure) were taken for modeling. Also, a blood sample was taken and total serum cholesterol in mmol/l was determined in one of two central laboratories with high standard of quality control. The cholesterol measurement was converted in mg/dl using the factor 38.667. Cholesterol was taken as an approximation to LDL blood levels due to the fact that laboratory tests on HDL/LDL were not available in 1218 of the participants, due to unsuccessful precipitations. Body height and weight were measured by medical staff. The resulting body mass index (BMI) was calculated by the study group and is part of the data set.

### Level 1 variables (individual level)

Age (in completed years, mean age: 45.6 years in men, 46.5 years in women) and BMI (as continuous variable, mean BMI: 26.3 kg/m^2 ^in men, 25.5 kg/m^2 ^in women) were centered about the grand mean (all values were converted so that the mean is 0).

We applied the so called 'Winkler-Index' to assess socioeconomic status (SES). This index is a three dimensional, additive, non-weighted social class index using education, occupation and household-income as indicators. This index is based on a short version of the 'Scheuch-Index', one of the few indices of its kind that has been validated. Each indicator ranges in points from 1 to 7, with 1 representing the lowest and 7 the highest status. The Winkler index can thus take values between 3 and 21 points [24]. Based on this score, 3 social classes with equally-sized ranges were defined. Lower SES ranges from 3 to 8 points, middle SES from 9 to 14 and upper SES from 15 to 21 points. The simple three-class model has the advantage that semantics matches everyday life perception of social classification.The variable 'education' was defined by highest academic/professional qualification. Monthly net household income with 11 categories served as basis for the 'income* variable and 'occupation' comprised 20 categories, which were subsumed in 5 groups (blue collar workers, self-employed persons including family workers, white collar workers, civil servants including professional soldiers and others [24].

Problems occur in assigning social class to the non-employed part of the German population; hence, the occupation of the person with the main household salary was used to define socioeconomic status of each person in the household. Married women most often provide a smaller portion of the household salary [25] and their status then is defined by the occupational status of the husband. For this reason, for women additional analyses were performed using the educational status – cut into 3 categories: lower, middle, upper SES – instead of the Winkler index to examine the degree of disagreement between the household based (husband based) SES definition of the index and the individual based educational status.

The occupational status of pensioners was defined by the last occupational activity. The variable SES was coded as dummy variable with lower SES as reference.

To measure status inconsistency, for all of the three SES-variables three categories were defined: category "low" comprising 1 and 2 points, category "medium" with 3, 4 and 5 points and category "high" comprising 6 and 7 points. If the SES of a person was classified by any combination of the categories "low" and "high" from SES indicators, this was considered as status inconsistency. Analyses were performed for both sexes separately.

### Level 2 variable (regional level)

Equivalence income was calculated on the basis of household income and number of persons in the respective household using the non-modified OECD-scale. The first adult was weighted with a factor of 1.0, additional adults with 0.7. Children up to 18 years were weighted with 0.5. The equivalence income thus calculated for all regions was 1427 DM (729.6 €).A per-capita income of less than 713 DM (364.6 €) was considered as relative poverty, affecting between 5.6 % (region 2) and 21.0 % (region 3) of participants of both sexes. This variable (see table 2) was centered about the grand mean and assesses a change of 1 % in the proportion of relative poverty in the corresponding models.

**Table 2:** Summary of the results from the multilevel models

	Men		Women	
Outcome variables	SES ^a^ (individual level)	Poverty Rate ^b^ (regional level)	SES ^a^ (individual level)	Poverty Rate ^b^ (regional level)
BMI	sign. association ^c^↓	(no association)^e^	sign. association^c^↓	weak association^d^↑
Systolic BP	sign. association^c^↓	weak association^d^↓	weak association^d^↓	(no association)^e^
Diastolic BP	weak association^d^↑	sign. association^c^↓	sign. association^c^↑	weak association^d^↓
Cholesterol	(no association)^e^	sign. association^c^↓	sign. association^c^↓	sign. association^c^↓

a) risk of upper SES as compared with lower SES

(↓ : lower risk in upper SES group; ↑ : higher risk in upper SES group)

b) risk associated with change in poverty rate of 1%

(↓ : decreasing risk with increasing poverty rate; ↑ : increasing risk with increasing poverty rate)

c) p < 0.05

d) p < 0.175

e) variable removed from the model due to exclusion criteria (α > 0.175)

### Statistical analysis

Multilevel modeling (individual level variables and group level variables are analysed simultaneously in one model) was used to examine the effects of individual and regional level variables, taking the cluster sampling approach into consideration, by applying the SAS procedure "Mixed". In the baseline model, only the variable age was included. In the full model, all additional variables according to the respective modeling were included (see additional file 1). Variables with α > 0,175 were removed stepwise as in a backward elimination (this means that the respective variable with the highest p-value is removed from the model) with the exception of the dummy variable SES, which was removed only when middle and upper SES did not fulfill the criterion (lower SES is reference). Thus the p-value for eliminating variables is within the range of 0.15 to 0.20, as recommended in Hosmer & Lemeshow for stepwise regression [26], p108.Baseline and final models are presented in tables 4 to 7 (see additional files 2,3,4,5). Analysis of residuals was performed only when there was a significant regional level variance in the final model.

### Sensitivity analyses

In a first step the base model (age only) was extended by including the aggregate level-variable 'relative poverty'. Then the three components of the social class index (educational level, income, profession) were added and considered separately and in all combinations. In a second step, the level 2 variable, 'relative poverty', defined by equivalence income was replaced with 'per-capita income' and then with an aggregated 'household income'-variable and 'unemployment-rate' calculated by means of self-reported data of participants. Additionally, the 'gross value added per inhabitant' from 1988 was taken as level 2 variable. This value ranges from 26,500 DM in Karlsruhe to 66,000 DM in Stuttgart (taken from an internal paper of the DHP-study group).

The German Cardiovascular Prevention Study was approved by the responsible ethics commission.

## Results

The total sample comprised N = 11,548 persons (5464 males, 6084 females). 528 cases (4.6 %) were deleted due to missing values. The analysis of missing data showed an arbitrary missing pattern. The maximum number of missing values was 361 for cholesterol (both genders). There is heterogeneity of mean cardiovascular risk factor levels at baseline [27] and also in terms of income inequality. The prevalence of lower SES varies in men from 10.8 % in region 1 to 24.1 % in region 3, and in women between 20.2 % in region 6 and 38.7 % in region 7 (table 1).

Data was analyzed for status inconsistencies that might be masked using the Winkler-Index. Inconsistencies were found in 4,5 % of participants (N = 494). They more often occur in females (5.2 %; N = 299) than in males (3,7 %; N = 195). As the results are only marginally affected, the respective data has not been removed.

Table 3 (see additional file 1) shows all variables that have been used for modeling and sensitivity analyses.

### Body mass index

#### Men

In the final model, different fixed individual effects were analyzed. The mean region-level score for men is 26.3 kg/m^2 ^on condition that the remaining predictors in the models are 0 (centered about grand mean) and with lower SES as reference. The model demonstrates that BMI increases with age (an increase of one year in age is accompanied by an increase of 0.07 kg/m^2 ^in BMI). The association with higher socioeconomic status is inconsistent, as BMI increases in middle SES but decreases in upper SES. The association of individual SES (upper class) with BMI level is statistically significant.

The level 2 variable 'relative poverty' (household income <713 DM), describing the fixed regional effect, has been removed because of the pre-defined exclusion criterion (p > 0.175). Social inequality at aggregate level thus cannot explain differences of BMI in men. Results show a statistically significant association of individual SES (upper class) on BMI level.

Examining random effects allows a comparison of individual and regional effects on total variance not explained by fixed effects: Most of total variance occurred at the individual level (estimates: 11.0 for individual vs. 0.17 for regional level). Regional level variance is small and not statistically significant. The proportion of total variance explained at regional level is 1.5 %.

#### Women

For the final model, all variables fulfill the inclusion criterion (p <= 0.175). The mean region-level score is 26.6 kg/m^2^. In women, BMI increases with age and decreases with higher socioeconomic status. A weak association of poverty with BMI is found. Most of the proportion of total variance is explained by individual level variance. Regional level variance is small and not significant and explains only 0.7 % of total variance for the final model (see additional file 2).

### Systolic blood pressure

In *men*, blood pressure increases with age and increasing BMI and decreases with higher socioeconomic status. A statistically significant effect of individual SES on systolic blood pressure can be shown. In women, high blood pressure increases with age and BMI level. Associations with SES groups are inconsistent (see additional file 3).

### Diastolic blood pressure

For men, a small but statistically significant effect of inequality on the aggregate level (variable „poverty") was found (see additional file 4). For women, results show an inverse association of diastolic blood pressure with SES.

### Residuals: Comparing the regions

**Figure 2:**
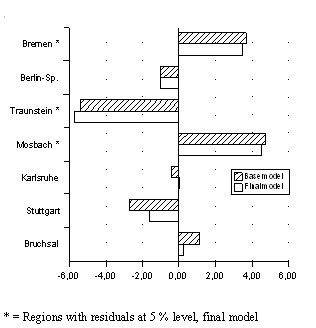
Residuals from multivariate models, systolic blood pressure, women.

Residuals are the differences between observed values and values predicted by the model, calculated as 'observed – predicted'. Residuals describe the variance not explained by the corresponding model. Analysis of residuals was performed only when there was a significant regional level variance in the final model. This only applied to the final model on systolic blood pressure for women. Blood pressure values varies from +4.5 mmHg in Mosbach to -5.7 mmHg in Traunstein, when the mean systolic blood pressure of all regions was set to 0 (see figure 2). In addition, differences can be observed for Bremen (+3.4 mmHg) and Stuttgart (-1.6 mmHg). All other regions lie close to the average.

### Serum cholesterol

#### Men

The variable poverty shows a statistically significant association with cholesterol level (see additional file 5). An increase of 1 % in the proportion of poverty results in a decrease of cholesterol by -0.58 mg/dl.

#### Women

A significant association of poverty with cholesterol level was found. Effects of SES are moving in opposite directions. The effect of BMI is small but significant.

To avoid possible over-adjustment, additional analyses were performed without the BMI variable. Results do not differ in a noteworthy manner.

### Educational status (women)

#### BMI

While estimates for middle SES (est. -1.49; p < 0.001) and upper SES (est. -3.01; p < 0.001) differ from the model using the Winkler index, the area level variable is not affected by the use of the educational status.

#### Systolic blood pressure

Again estimates for SES differ: middle SES (est.: 1.06; p < 0.001) and upper SES (est. -1.56; p < 0.001). The level 2 variable has been removed as in the original model.

### Sensitivity analyses

To test the sensitivity of the results towards modeling assumptions, different models for the variables BMI and diastolic blood pressure were performed. The results of the level 2 variable 'relative poverty' do not change in any model (see additional file 6). Thus, a possible composite effect seems to be very small in both models. Removing the level 2 variable from corresponding models does not affect the level 1 variables in any appreciable way.

In a second step, the level 2 variable 'relative poverty' defined by equivalence income was replaced with other variables. Comparing the level 2 variables, results are rather similar, with the exception of the unemployment-rate and the gross value added per inhabitant (see additional file 7).

## Discussion

Health inequalities have been studied in Germany for some time, and the results are very clear: Morbidity and mortality increase strongly with decreasing socio-economic status [28]. These observations are very similar to those in other Western European countries [29]. Little information is available concerning the regional aspects of health inequalities, i.e. the differences in morbidity and mortality between regions characterized by indicators of socio-economic status. Also, to date no regional deprivation score has been developed.

There is a long tradition of using these scores, mainly in the UK. Well known are, for example, the Townsend Score, the Carstairs Index, the Jarman Score, the Breadline Score, and the Index of Multiple Deprivation [30-33]. In the UK, the health care system is largely organized on the regional level; this is why these deprivation scores carry very practical importance.

Lynch and colleagues [34] reviewed 98 aggregate and multilevel studies examining associations of income inequality with health. For the US, a robust association was found between income inequalities and health outcomes in aggregated data at the state level. In multilevel studies, results are most consistent at the state level, while results at other levels are inconsistent. For other countries, both aggregated and multilevel studies suggest a small or no effect, except in the United Kingdom. Additionally, time trends in income inequality and mortality show little congruence at the national level in the US [35].

Multilevel analyses are rarely conducted in Germany. To date, there seems to be just one published study; and it is difficult to access for the international audience, as it is published in German [36]. The analyses are based on interviews with about 400 adults from 38 city districts in Cologne. The results show that morbidity is lower in the upper status districts, and that much of this difference is explained by individual social status. Just about 5% of the variance is explained by regional effects; this effect is statistically significant (p < 0.05), but still rather small.

In our study, potential effects of inequality at regional level on the cardiovascular risk factors – BMI, systolic and diastolic blood pressure, as well as serum cholesterol – were analyzed using mixed models. The level 1 variables, age, SES, body mass index, and high serum cholesterol, respectively, and the level 2 variable, poverty, were included as explanatory variables. The effects of area on each risk factor were examined separately. All analyses were performed separately for males and females. The analysis addresses directly the question as to whether there is evidence for an association between income inequality and risk factor level at area level. Fixed effects show an increase of the respective risk factor level with age for all models. Also, the body mass index is significantly associated with risk factor levels of systolic and diastolic blood pressure and serum cholesterol.

An association of socioeconomic status with risk factors has been found in most models. SES has no significant effect on cholesterol and diastolic blood pressure in men. The level 2 variable, poverty, has been removed from 2 of the 8 models. A statistically significant association has been found with diastolic blood pressure and cholesterol in men, and with cholesterol in women, indicating that regional inequality (poverty) as explanatory variable plays a role regarding risk factor levels. A weak association has been found for systolic blood pressure in men and BMI and diastolic blood pressure in women.

Gini Coefficients (SAS code by Philip N. Cohen [37]) calculated on the basis of the income variable with 7 categories showed differences between regions too small to test the hypothesis. Consequently, regional differences in risk factor levels cannot be explained by income distribution as measured with this coefficient. Instead, the variable 'relative poverty' has been defined to describe inequality at regional level.Analysis of residuals (systolic blood pressure in women) shows that "healthy" and "unhealthy" regions can be identified. The region of Traunstein can be defined as a "healthy" region and Moosbach as the "un-healthiest" region in this study, if the effects in the corresponding model (figure 2) are considered. It has to be pointed out, however, that these differences between regions are based on other effects than relative poverty.

The finding that individual socio-economic status is more relevant to health inequalities than regional differences of per-capita income points to the fact that these two levels of stress have different impacts on health: Individual-level stressors influence the health status of the individuals exposed to these stressors very directly. Ecological-level stressors could add an extra burden on health, but the effect of this extra burden is much more indirect. Future studies should aim at analyzing the interactions between these different stressors in more detail.

Weaknesses of this study: The results are based on the baseline data of 6 intervention regions of a nation-wide cross-sectional health survey conducted during the mid-1980s. Besides methodological restrictions due to the study design (cross-sectional), the age of the data does not allow to draw conclusions with regard to the current situation in Germany. This situation has changed considerably due to the reunification and the migration of labor from east to west and as a consequence of aging populations in many regions of the east. The steep rise in unemployment, flexible work arrangements, and fixed-term contracts during the past few years are other important changes potentially affecting the health status of the population.

The present study used predefined intervention regions for analysis. Hence, a definition of smaller or larger regional units was not feasible.

Using the Winkler index to define SES might bias the information on social status. Reasons are status inconsistencies and the inclusion of unemployed persons – reasons that are especially relevant to women. In the latter case, the position of the husband in the occupational system has been used to define one of the three indicators of the index. Hence, additional analyses were performed omitting inconsistent data and with educational status as alternative indicator of SES. Comparisons show that the Winkler index bias results only marginally when inconsistent data are not removed.

Another point in coding the index might be an object of discussion: The use of the occupational status of the person earning the main household salary. Thus the status of unemployed married women, but also of married women in part-time or comparably low paid full time employment, is partially defined by the status of their husbands. This approach can bias results.

The corresponding analysis in women showed stronger differences, using educational status instead of the index, but again results changed only to a small degree.Strengths of this study: Data for the mid-1980s is available only from few statistical departments of the German federal states. Therefore the German Cardiovascular Prevention Study is an important data base to describe population health in the 1980s. The results of this paper can serve as a basis to analyze the temporal changes in Germany over the past 25 years.

Data of the German Cardiovascular Prevention Study allow for an analysis of individual and geographical variations in cardiovascular risk factors as predictors of cardiovascular morbidity and mortality. The intervention regions examined are characterized by differences in the size of cities, in the proportion of blue collar workers, in average income, etc. As a whole, however, the pooled intervention regions are similar in characteristics to former West Germany. Using data from the time before re-unification means that we do not have to deal with major changes in economic situation, and possibly cardiovascular risk profiles, within a very short time period. Excluding non-German residents avoids bias, as migrants often "bring along" lower cardiovascular risk factor levels from their countries of origin [38].

## Conclusion

During the mid-1980s, regional effects of social inequality on cardiovascular health played a minor role in Germany compared to individual effects. The fact that individual measures of cardiovascular risk factors are largely explained by individual measures of social status stress the importance of interventions focusing on persons with low socio-economic status. It is important to point out, though, that regions characterized by high income inequalities show an increased risk of high blood pressure and high levels of total serum cholesterol. These regional effects are hard to detect, and they are rather small, but they still give some support to the hypothesis that income inequality is a risk factor in its own right.

## Competing interests

The author(s) declare that they have no competing interests.

## Authors' contributions

OR had the initial idea for this paper, which was subsequently modified in discussions with JB and AM. JB conducted the data analysis. Results were interpreted by all three authors. JB and OR wrote the initial draft of the manuscript which was revised by all authors. All authors read and approved the final manuscript.

## Pre-publication history

The pre-publication history for this paper can be accessed here:



## Supplementary Material

Additional file 1 Multilevel model variables. Click here for file

Additional file 2 Results of multilevel models for body mass index (BMI, kg/m^2^). Click here for file

Additional file 3 Results of multilevel models for systolic blood pressure (mm/Hg). Click here for file

Additional file 4 Results of multilevel models for diastolic blood pressure (mm/Hg). Click here for file

Additional file 5 Results of multilevel models for cholesterol (mg/dl). Click here for file

Additional file 6 Results of sensitivity analyses (level 1-variables). Click here for file

Additional file 7 Results of sensitivity analyses (level 2-variables). Click here for file
