# Supplementary material for: Health inequalities in Germany: do regional-level variables explain differentials in cardiovascular risk?
Source: BMC Public Health. 2007 Jul 1;7:132. doi: 10.1186/1471-2458-7-132 (PMC1934354; doi:10.1186/1471-2458-7-132)
Supplement: Additional file 1 — Multilevel model variables. [file 1471-2458-7-132-S1.doc]

**Additional file 1:** Multilevel model variables

| ***Explanatory variables*** | Unit(s) | Procedures | Level | Measure |
| --- | --- | --- | --- | --- |
| Age | completed years | centered about grand mean | 1 | mean |
| Socio-economic status (SES) | dummy variable |  | 1 | - |
| Body-mass-index | continuous | centered about grand mean | 1 | mean |
| Relative poverty (regional level) | < 50 % of mean equivalence income | centered about grand mean ^a^ | 2 | percent |
| ***Class-variable*** |  |  |  |  |
| Region | region 1 to region 7 |  |  | - |
| ***Outcome variables*** |  |  |  |  |
| Body-mass-index | continuous |  |  | mean |
| Systolic blood pressure | continuous |  |  | mean |
| Diastolic blood pressure | continuous |  |  | mean |
| Cholesterol | continuous |  |  | mean |
| ***Additional variables for*** ***sensitivity analyses*** | |  |  |  |
| income | categorical |  | 1 |  |
| education | categorical |  | 1 |  |
| professional status | categorical |  | 1 |  |
| relative poverty II (regional level) | < 50 % of mean per-capita income | centered about grand mean ^a^ | 2 | percent |
| relative poverty III (regional level) | household income < 2000 DM | centered about grand mean ^a^ | 2 | percent |
| unemployment rate |  | centered about grand mean | 2 | percent |
| gross value added per inhabitant | categorical | centered about grand mean ^b^ | 2 |  |

a) results express a change of 1% in the percentage of the population below the poverty line

b) results express a change of 1000 DM of the gross value added per inhabitant
