# Supplementary material for: Health inequalities in Germany: do regional-level variables explain differentials in cardiovascular risk?
Source: BMC Public Health. 2007 Jul 1;7:132. doi: 10.1186/1471-2458-7-132 (PMC1934354; doi:10.1186/1471-2458-7-132)
Supplement: Additional file 2 — Results of multilevel models for body mass index (BMI, kg/m2). [file 1471-2458-7-132-S2.doc]

**Additional file 2:** Results of multilevel models for body mass index (BMI, kg/m^2^)

|  | Men (n = 5,234) | | | | | | Women (n = 5,786) | | | | | |
| --- | --- | --- | --- | --- | --- | --- | --- | --- | --- | --- | --- | --- |
|  | Base Model | | | Final Model | | | Base Model | | | Final Model | | |
|  | Est. | SE | P | Est. | SE | P | Est. | SE | P | Est. | SE | P |
| *Fixed effects* |  |  |  |  |  |  |  |  |  |  |  |  |
| Intercept (constant) | 26.3 | 0.17 | < .001 | 26.3 | 0.210 | < .001 | 25.6 | 0.21 | < .001 | 26.6 | 0.164 | < .001 |
| Age (individual) | 0.07 | 0.004 | < .001 | 0.07 | 0.004 | < .001 | 0.13 | 0.004 | < .001 | 0.12 | 0.004 | < .001 |
| Middle SES (individual) |  |  |  | 0.15 | 0.128 | 0.240 |  |  |  | -1.13 | 0.127 | < .001 |
| Upper SES (individual) |  |  |  | -0.30 | 0.148 | 0.040 |  |  |  | -2.36 | 0.172 | < .001 |
| Poverty (regional) |  |  |  | - | - | - |  |  |  | 0.05 | 0.028 | 0.122 |
| *Random effects* |  |  |  |  |  |  |  |  |  |  |  |  |
| Level 1 (individual) | 11.00 | 0.22 | < .001 | 11.00 | 0.22 | < .001 | 17.56 | 0.33 | < .001 | 17.01 | 0.316 | < .001 |
| Level 2 (regional) | 0.18 | 0.12 | 0.057 | 0.17 | 0.11 | 0.059 | 0.30 | 0.18 | 0.053 | 0.112 | 0.085 | 0.093 |
| How to read the table (e.g. men, final model): The intercept (column “est”) shows the mean BMI in kg/m^2^ (26.3 for all regions) if all predictor variables in the corresponding model are set to 0. Age = 0 (centered about grand mean) is the mean age of men from all regions (45.6 years). If age increases by one year (that is from 45.6 to 46.6), the BMI increases by 0.07 kg/m^2^). For "upper SES", the BMI decreases by 0.3 kg/m^2^. | | | | | | | | | | | | |
