# Supplementary material for: Health inequalities in Germany: do regional-level variables explain differentials in cardiovascular risk?
Source: BMC Public Health. 2007 Jul 1;7:132. doi: 10.1186/1471-2458-7-132 (PMC1934354; doi:10.1186/1471-2458-7-132)
Supplement: Additional file 3 — Results of multilevel models for systolic blood pressure (mm/Hg). [file 1471-2458-7-132-S3.doc]

| **Additional file 3:** Results of multilevel models for systolic blood pressure (mm/Hg) | | | | | | | | | | | | |
| --- | --- | --- | --- | --- | --- | --- | --- | --- | --- | --- | --- | --- |
|  | Men  N = 5234 | | | | | | Women  N = 5786 | | | | | |
|  | Base | | | Final | | | Base | | | Final | | |
|  | Est. | SE | P | Est. | SE | P | Est. | SE | P | Est. | SE | P |
| *Fixed effects* |  |  |  |  |  |  |  |  |  |  |  |  |
| Intercept (constant) | 135.3 | 1.13 | < .001 | 136.3 | 1.09 | < .001 | 130.4 | 1.38 | < .001 | 129.8 | 1.38 | < .001 |
| Age (individual) | 0.48 | 0.02 | < .001 | 0.40 | 0.02 | < .001 | 0.82 | 0.02 | < .001 | 0.67 | 0.02 | < .001 |
| Middle SES (individual) |  |  |  | -0.96 | 0.67 | 0.161 |  |  |  | 1.41 | 0.54 | 0.009 |
| Upper SES (individual) |  |  |  | -2.51 | 0.77 | < .001 |  |  |  | -1.20 | 0.74 | 0.104 |
| Poverty (regional) |  |  |  | -0.33 | 0.20 | 0.158 |  |  |  | - | - | - |
| BMI |  |  |  | 1.11 | 0,07 | < .001 |  |  |  | 1.16 | 0.06 | < .001 |
| *Random effects* |  |  |  |  |  |  |  |  |  |  |  |  |
| Level 1 (individual) | 309.5 | 6.06 | < .001 | 295.3 | 5.78 | < .001 | 326.7 | 6.08 | < .001 | 302.0 | 5.62 | < .001 |
| Level 2 (regional) | 8.40 | 5.11 | 0.050 | 5.87 | 4.00 | 0.071 | 12.90 | 7.73 | 0.048 | 11.9 | 7.1 | 0.048 |
|  | | | | | | | | | | | | |
