# Supplementary material for: Health inequalities in Germany: do regional-level variables explain differentials in cardiovascular risk?
Source: BMC Public Health. 2007 Jul 1;7:132. doi: 10.1186/1471-2458-7-132 (PMC1934354; doi:10.1186/1471-2458-7-132)
Supplement: Additional file 4 — Results of multilevel models for diastolic blood pressure (mm/Hg). [file 1471-2458-7-132-S4.doc]

**Additional file 4:** Results of multilevel models for diastolic blood pressure (mm/Hg)

|  | Men (n = 5,234) | | | | | | Women (n = 5,786) | | | | | |
| --- | --- | --- | --- | --- | --- | --- | --- | --- | --- | --- | --- | --- |
|  | Base Model | | | Final Model | | | Base Model | | | Final Model | | |
|  | Est. | SE | P | Est. | SE | P | Est. | SE | P | Est. | SE | P |
| *Fixed effects* |  |  |  |  |  |  |  |  |  |  |  |  |
| Intercept (constant) | 84.4 | 0.98 | < .001 | 83.6 | 0.61 | < .001 | 80.4 | 0.86 | < .001 | 79.5 | 0.72 | < .001 |
| Age (individual) | 0.14 | 0.01 | < .001 | 0.07 | 0.01 | < .001 | 0.27 | 0.01 | < .001 | 0.17 | 0.01 | < .001 |
| Middle SES (individual) |  |  |  | 0.74 | 0.43 | 0.086 |  |  |  | 1.34 | 0.34 | < .001 |
| Upper SES (individual) |  |  |  | 0.78 | 0.50 | 0.117 |  |  |  | 1.06 | 0.47 | 0.023 |
| BMI (individual) |  |  |  | 1.01 | 0.05 | < .001 |  |  |  | 0.75 | 0.03 | < .001 |
| Poverty (regional) |  |  |  | -0.43 | 0.10 | 0.008 |  |  |  | -0.29 | 0.14 | 0.092 |
| *Random effects* |  |  |  |  |  |  |  |  |  |  |  |  |
| Level 1 (individual) | 136.9 | 2.68 | < .001 | 125.7 | 2.46 | < .001 | 129.6 | 2.41 | < .001 | 119.8 | 2.23 | < .001 |
| Level 2 (regional) | 6.56 | 3.88 | 0.046 | 1.54 | 1.08 | 0.077 | 4.93 | 2.93 | 0.046 | 3.11 | 2.06 | 0.066 |
