# Supplementary material for: Health inequalities in Germany: do regional-level variables explain differentials in cardiovascular risk?
Source: BMC Public Health. 2007 Jul 1;7:132. doi: 10.1186/1471-2458-7-132 (PMC1934354; doi:10.1186/1471-2458-7-132)
Supplement: Additional file 5 — Results of multilevel models for cholesterol (mg/dl). [file 1471-2458-7-132-S5.doc]

**Additional file 5:** Results of multilevel models for cholesterol (mg/dl)

|  | Men (n = 5,234) | | | | | | Women (n = 5,786) | | | | | |
| --- | --- | --- | --- | --- | --- | --- | --- | --- | --- | --- | --- | --- |
|  | Base Model | | | Final Model | | | Base Model | | | Final Model | | |
|  | Est. | SE | P | Est. | SE | P | Est. | SE | P | Est. | SE | P |
| *Fixed effects* |  |  |  |  |  |  |  |  |  |  |  |  |
| Intercept (constant) | 233.3 | 1.58 | < .001 | 233.2 | 1.06 | < .001 | 233.5 | 1.07 | < .001 | 232.6 | 1.17 | < .001 |
| Age (individual) | 0.96 | 0.05 | < .001 | 0.78 | 0.05 | < .001 | 1.97 | 0.05 | < .001 | 1.87 | 0.05 | < .001 |
| Middle SES (individual) |  |  |  | - | - | - |  |  |  | 2.86 | 1.33 | 0.032 |
| Upper SES (individual) |  |  |  | - | - | - |  |  |  | -3.36 | 1.83 | 0.066 |
| BMI (individual) |  |  |  | 2.43 | 0.19 | < .001 |  |  |  | 0.76 | 0.14 | < .001 |
| Poverty (regional) |  |  |  | -0.58 | 0.21 | 0.043 |  |  |  | -0.39 | 0.14 | 0.039 |
| *Random effects* |  |  |  |  |  |  |  |  |  |  |  |  |
| Level 1 (individual) | 2078.4 | 40.66 | < .001 | 2013.9 | 39.4 | < .001 | 1873.1 | 34.85 | < .001 | 1858.5 | 34.59 | < .001 |
| Level 2 (regional) | 14.4 | 10.29 | 0.076 | 5.11 | 5.48 | 0.175 | 5.65 | 4.51 | 0.105 | 1.41 | 2.41 | 0.279 |
