# Supplementary material for: Health inequalities in Germany: do regional-level variables explain differentials in cardiovascular risk?
Source: BMC Public Health. 2007 Jul 1;7:132. doi: 10.1186/1471-2458-7-132 (PMC1934354; doi:10.1186/1471-2458-7-132)
Supplement: Additional file 6 — Results of sensitivity analyses (level 1-variables). [file 1471-2458-7-132-S6.doc]

| **Additional file 6:** Results of sensitivity analyses (level 1-variables) | | | | | | | | | | | | |
| --- | --- | --- | --- | --- | --- | --- | --- | --- | --- | --- | --- | --- |
|  | Men  N = 5234 | | | | | | Women  N = 5786 | | | | | |
|  | BMI  estimates of relative poverty | | | diastolic blood pressure | | | BMI | | | diastolic blood pressure | | |
| *base model* | Est. | SE | P | Est. | SE | P | Est. | SE | P | Est. | SE | P |
| *age and relative poverty included* | 0.02 | 0.04 | 0.62 | -0.41 | 0.13 | 0.02 | 0.08 | 0.03 | 0.07 | -0.25 | 0.15 | 0.17 |
| + SES variable | 0.02 | 0.04 | 0.65 | -0.41 | 0.12 | 0.02 | 0.05 | 0.03 | 0.12 | -0.25 | 0.15 | 0.17 |
| + income instead of SES* | 0.02 | 0.04 | 0.61 | -0.40 | 0.12 | 0.02 | 0.07 | 0.03 | 0.09 | -0.24 | 0.15 | 0.17 |
| + education instead of SES* | 0.01 | 0.04 | 0.72 | -0.42 | 0.13 | 0.02 | 0.05 | 0.03 | 0.13 | -0.26 | 0.15 | 0.14 |
| + profession instead of SES* | 0.02 | 0.04 | 0.60 | -0.41 | 0.13 | 0.02 | 0.05 | 0.03 | 0.10 | -0.25 | 0.15 | 0.15 |
| + income and education* ... | 0.01 | 0.04 | 0.63 | -0.41 | 0.13 | 0.02 | 0.05 | 0.03 | 0.14 | -0.26 | 0.15 | 0.15 |
| + income and profession*... | 0.03 | 0.03 | 0.50 | -0.40 | 0.12 | 0.02 | 0.05 | 0.03 | 0.10 | -0.25 | 0.15 | 0.16 |
| + education and profession* ... | 0.02 | 0.04 | 0.66 | -0.41 | 0.13 | 0.02 | 0.04 | 0.02 | 0.15 | -0.27 | 0.15 | 0.14 |
| + income,education, profession* ... | 0.02 | 0.03 | 0.55 | -0.41 | 0.13 | 0.02 | 0.04 | 0.02 | 0.14 | -0.26 | 0.15 | 0.14 |
| * each with 7 categories, not centered | | | | | | | | | | | | |
