# Supplementary material for: Health inequalities in Germany: do regional-level variables explain differentials in cardiovascular risk?
Source: BMC Public Health. 2007 Jul 1;7:132. doi: 10.1186/1471-2458-7-132 (PMC1934354; doi:10.1186/1471-2458-7-132)
Supplement: Additional file 7 — Results of sensitivity analyses (level 2-variables). [file 1471-2458-7-132-S7.doc]

| **Additional file 7:** Results of sensitivity analyses (level 2-variables) | | | | | | | | | | | | |
| --- | --- | --- | --- | --- | --- | --- | --- | --- | --- | --- | --- | --- |
|  | Men  N = 5234 | | | | | | Women  N = 5786 | | | | | |
|  | BMI | | | diastolic blood pressure | | | BMI | | | diastolic blood pressure | | |
| final models  with ... | Est. | SE | P | Est. | SE | P | Est. | SE | P | Est. | SE | P |
| *equivalence income** | *0.02* | *0.04* | *0.64* | *-0.43* | *0.10* | *0.01* | *0.05* | *0.03* | *0.12* | *-0.29* | *0.14* | *0.09* |
| household income* | 0.01 | 0.04 | 0.88 | -0.42 | 0.15 | 0.04 | 0.05 | 0.03 | 0.24 | -0.15 | 0.20 | 0.50 |
| per-capita income* | 0.02 | 0.02 | 0.39 | -0.27 | 0.09 | 0.03 | 0.04 | 0.02 | 0.11 | -0.20 | 0.09 | 0.08 |
| unemployment rate | -0.01 | 0.16 | 0.97 | -0.83 | 0.86 | 0.38 | 0.07 | 0.16 | 0.67 | -0.17 | 0.83 | 0.85 |
| gross value added per inhabitant** | -0.02 | 0.01 | 0.03 | 0.01 | 0.07 | 0.86 | -0.02 | 0.01 | 0.02 | 0.005 | 0.07 | 0.95 |
| *Townsend index could not be used, because necessary data are not available*  ** estimates describe a change of 1 % in relative poverty*  *** estimates describe a change of 1000 DM gross value added per inhabitant* | | | | | | | | | | | | |
